# Development and evaluation of an ^18^F-labeled nanobody to target SARS-CoV-2's spike protein

**DOI:** 10.3389/fnume.2022.1033697

**Published:** 2022-11-23

**Authors:** Sara Lopes van den Broek, Rocío García-Vázquez, Ida Vang Andersen, Guillermo Valenzuela-Nieto, Vladimir Shalgunov, Umberto M. Battisti, David Schwefel, Naphak Modhiran, Vasko Kramer, Yorka Cheuquemilla, Ronald Jara, Constanza Salinas-Varas, Alberto A. Amarilla, Daniel Watterson, Alejandro Rojas-Fernandez, Matthias M. Herth

**Affiliations:** ^1^ Department of Drug Design and Pharmacology, Faculty of Health and Medical Sciences, University of Copenhagen, Copenhagen, Denmark; ^2^ Institute of Medicine, Faculty of Medicine & Center for Interdisciplinary Studies on the Nervous System, CISNE, Universidad Austral de Chile, Valdivia, Chile; ^3^ Institute of Medical Physics and Biophysics, Charité – Universitätsmedizin Berlin, Corporate Member of Freie Universität Berlin and Humboldt-Universität zu Berlin, Berlin, Germany; ^4^ School of Chemistry and Molecular Biosciences, University of Queensland, St Lucia, QLD, Australia; ^5^ Australian Institute for Bioengineering and Nanotechnology, The University of Queensland, St Lucia, QLD, Australia; ^6^ Positronpharma SA, Providencia, Santiago, Chile; ^7^ Berking Biotechnology, Valdivia, Chile; ^8^ Department of Clinical Physiology, Nuclear Medicine & PET, Rigshospitalet Copenhagen University Hospital, Copenhagen, Denmark

**Keywords:** SARS-CoV-2, COVID-19, tetrazine ligation, PET, nanobodies, biodistribution, alpaca

## Abstract

COVID-19, caused by the SARS-CoV-2 virus, has become a global pandemic that is still present after more than two years. COVID-19 is mainly known as a respiratory disease that can cause long-term consequences referred to as long COVID. Molecular imaging of SARS-CoV-2 in COVID-19 patients would be a powerful tool for studying the pathological mechanisms and viral load in different organs, providing insights into the disease and the origin of long-term consequences and assessing the effectiveness of potential COVID-19 treatments. Current diagnostic methods used in the clinic do not allow direct imaging of SARS-CoV-2. In this work, a nanobody (NB) – a small, engineered protein derived from alpacas – and an Fc-fused NB which selectively target the SARS-CoV-2 Spike protein were developed as imaging agents for positron emission tomography (PET). We used the tetrazine ligation to ^18^F-label the NB under mild conditions once the NBs were successfully modified with *trans-*cyclooctenes (TCOs). We confirmed binding to the Spike protein by SDS-PAGE. Dynamic PET scans in rats showed excretion through the liver for both constructs. Future work will evaluate *in vivo* binding to the Spike protein with our radioligands.

## Introduction

The outbreak of the coronavirus disease 2019 (COVID-19) in Wuhan (China) in December 2019 rapidly developed into a global pandemic resulting in a significant public health disaster (1, 2). To date, COVID-19 has caused over six million deaths and infected hundreds of millions worldwide (3). The disease is caused by the severe acute respiratory syndrome coronavirus 2 (SARS-CoV-2) and is mainly known for its respiratory symptoms. However, many patients develop severe symptoms such as acute respiratory distress syndrome, multisystem inflammatory syndrome, fulminant myocarditis (4, 5), arterial and venous thromboembolism that can lead to lethal blood clots (6), hematological disorders, or respiratory and neurological conditions (7–12). These complications are an important concern for the management of COVID-19 and may be explained by the virus' ability to infect relevant organs (bone marrow, lymph nodes, heart, brain).

Despite the rapid development of vaccines and preventive measures against the viral spread and disease progression, COVID-19 still affects the lives of many people around the globe (13–15). This is due to several factors, such as the rise of new virus mutations and differences in vaccine susceptibility between individuals (16–20). COVID-19 is expected to become an endemic infectious disease; therefore, further insight into the virus is highly valuable. Imaging SARS-CoV-2 in COVID-19 patients will improve our understanding of the pathological mechanisms underlying the disease and its complications. Positron Emission Tomography (PET) is highly suitable for this purpose since it is a nuclear molecular imaging method that allows visualization and quantification of biological processes *in vivo* (21–25). It can also be used to assess the effectiveness of potential COVID-19 treatments (26, 27). This will decrease the time needed to develop a drug, which is especially urgent during this global pandemic that has been amongst us for over two years. Existing imaging methods currently used in the clinic can visualize the effects of SARS-CoV-2 infection but not the virus itself (28, 29).

SARS-CoV-2 is a single-stranded RNA virus whose particles are covered with 9–12 nm long Spike (S) protein (2). This protein plays a key role in the attachment of the virus to its host cell and entry to establish viral infection. The receptor-binding domain (RBD) of the Spike protein interacts with the angiotensin-converting enzyme 2 (ACE 2) receptor on the host cell surface, which, after multiple steps, results in membrane fusion (30). High affinity compounds that block Spike-ACE2 interaction can also be a valuable tool for imaging diagnostics to monitor in real-time viral dissemination in the body (23, 31, 32). Single-domain antibodies, also known as nanobodies (NB), have a high potential to be used as drugs and imaging agents since they often have high target selectivity, stability and tissue penetration capacity. Additionally, they are more stable and more accessible to produce than conventional antibodies (Abs). NBs can be derived from camelids, e.g., alpacas, as they, in addition to conventional Abs, can produce heavy chain only Abs (HCAbs). Lymphocytes producting HCAbs can be isolated from the blood, and the variable regions or VHH domain can then be amplified from mRNA and further selected and cloned for recombinated production. Isolated VHH are also known as nanobodies (NBs, SANOFI) (33–39). We have recently isolated an alpaca-derived NB – W25 - that selectively targets the Spike protein with an affinity of approximately 300 pM. This high affinity NB shows a high neutralizing effect of SARS-Cov-2, has high stability and can be produced at large scale and therefore has great potential for clinical purposes (38, 40).

**Figure 1:**
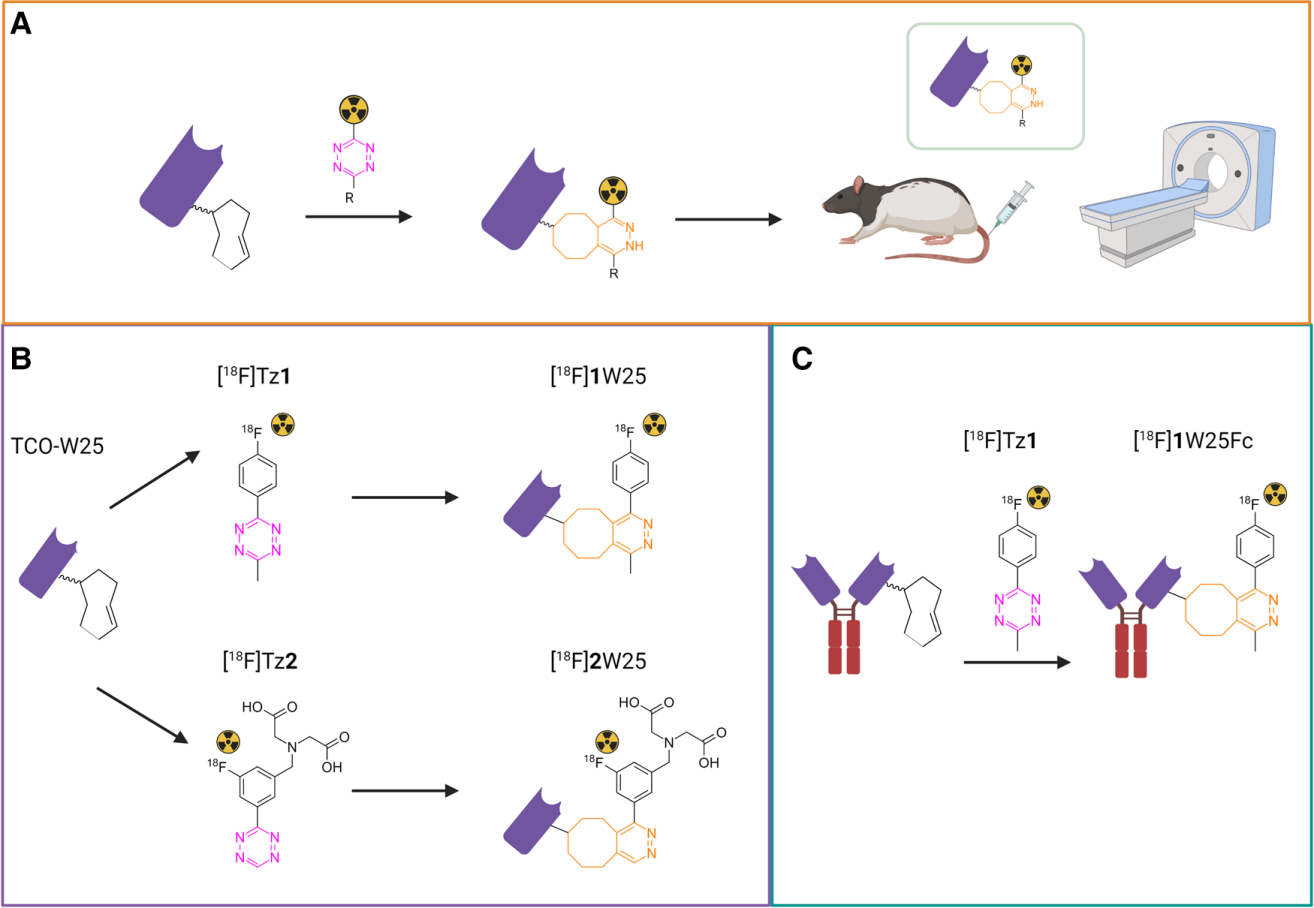
(**A**) study outline. The protein is first modified with TCO and radiolabeled with an ^18^F-Tz. The radiolabeled protein is injected intraveneously (i.v.) into rats and evaluated by PET. (**B**) Labeling of TCO-W25 with [^18^F]Tz**1** and [^18^F]Tz**2** using the tetrazine ligation (**C**) Labeling of TCO-W25Fc with [^18^F]Tz**1**.

In this study, we used NB W25 and its Fc-fused alternative – W25Fc – to develop tracers for positron emission tomography (PET) and image SARS-CoV-2 *via* its Spike protein. For this, we used tetrazine ligation since it labels the protein under very mild conditions (41–43). The tetrazine ligation is an inverse-electron demand Diels Alder (IEDDA) click reaction between a *trans*-cyclooctene (TCO) and a tetrazine (Tz) and followed by a retro-Diels Alder reaction. The ligation is ultrafast with rate constants >50000 M^−1^s^−1^ and bioorthogonal (42–49). We investigated if the tetrazine ligation could be used to label our NBs without purification and isolated in high molar activity (A_m_). This would be highly beneficial as it could lead to doses within the microdose scale (<100 μg NB per patient). Microdosing increases the safety of the imaging agent significantly, avoiding large, expensive, and time-consuming toxicology studies (50, 51). In our approach, we decided to conjugate TCOs to the protein and then ^18^F-label it with a Tz. The study outline is displayed in Figure 1A.

## Results and discussion

### Protein modifications and TCO quantification

The W25 NB displays a subnanomolar affinity for the Spike protein of SARS-CoV-2 and is a powerful neutralizing measure against SARS-CoV-2 infection in cells. We observed that cells transfected with GFP-tagged Spike show dynamic cycling of the Spike protein between the cell membrane (Figure 1A), thus we hypothesize that live H1299 cells - a lung carcinoma cell line - could capture the W25 NB in an active manner from the culture media into the surface of the cell membrane and also take up the W25 NB into endosomal compartments. In order to test this hypothesis, H1299 cells transfected with GFP-Spike were exposed for 1 h to the Myc-tagged W25 NB and added to the culture media. After incubation, cells were washed, fixed and analyzed by immunofluorescence. We confirmed that GFP-Spike cells trapped W25 *in vivo*, while transfected H1299 cells were unable to take up W25. Therefore, we demonstrated an active uptake of W25 in cells expressing the Spike protein of SARS-CoV-2 and we presume this NB could display great potential for *in vivo* imaging of infected tissue by PET (Figure 1B).

**Figure 2:**
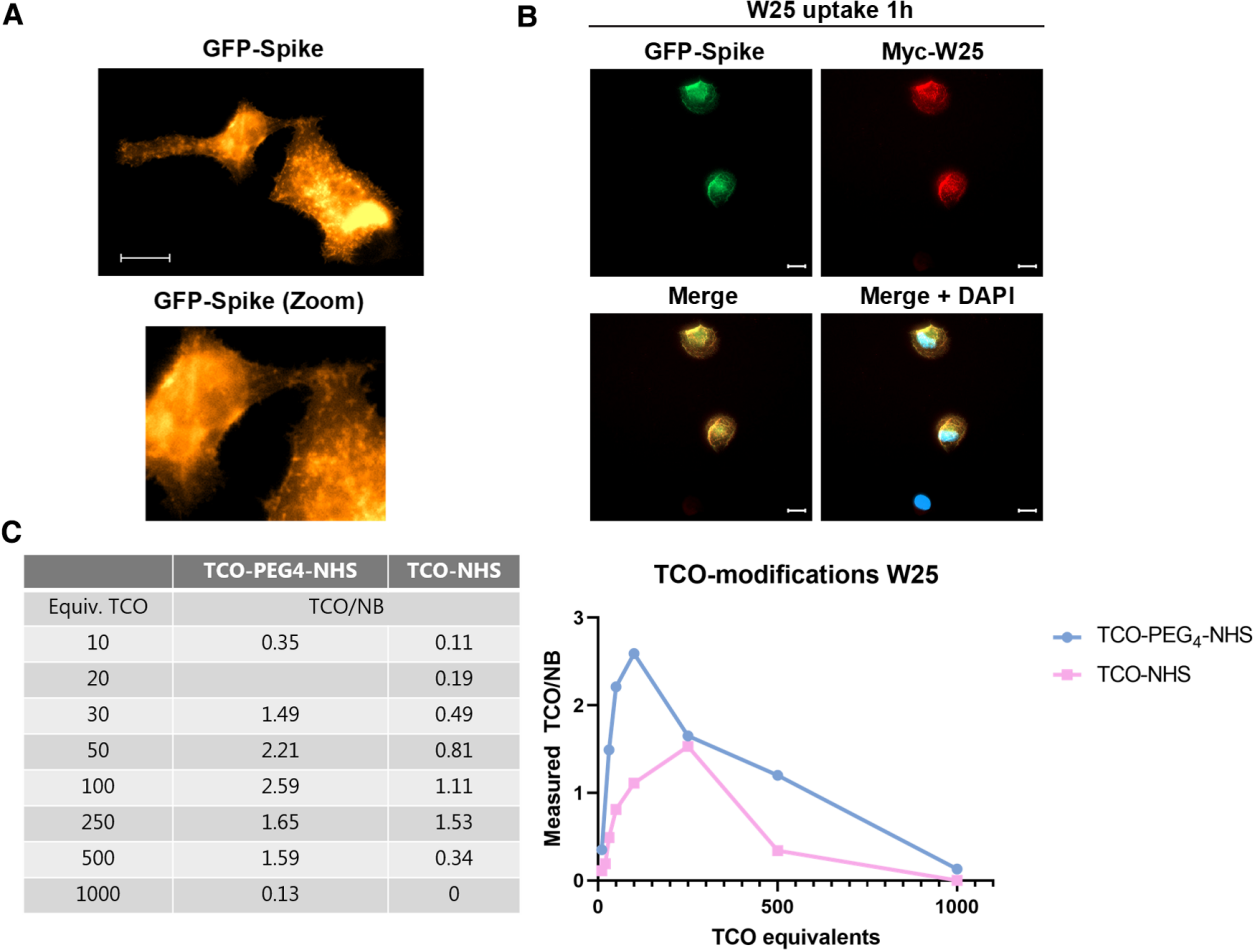
W25 binding to spike in cells and TCO modifications of W25 using TCO-PEG_4_-NHS and TCO-NHS ester. (**A**) H1299 cells were transfected with GFP-Spike (gold) and after 24 h the cells were image *in vivo* in a Celldiscoverer 7 automatic microscope. (**B**) H1299 cells transfected with GFP-Spike (Green) of SARS-CoV-2 were incubated 1 h with 1 μg/ml of myc-tagged W25 directly after the cells were washed, fixed and immunostained (myc-W25 in red), nucleus were stained with DAPI to unveil transfected cells (blue) scale bars are 20 um. (**C**) TCO modifications of W25 using TCO-PEG_4_-NHS and TCO-NHS ester. A maximum of 2.59 TCOs/NB was obtained using TCO-PEG_4_-NHS ester, and a maximum of 1.53 TCO/NB was obtained with TCO-NHS ester.

Further, W25 was modified with TCO using non-site-specific conjugation to lysine residues (52, 53). The NB contained 6 lysine residues, with one located in the antigen-binding region (complementarity-determining region (CDR)). Two different TCOs were evaluated for conjugation: TCO-PEG_4_-NHS ester and TCO-NHS ester. The number of reactive TCOs was determined by titration according to previously described protocols (54). The relationship between the amount of TCO taken for conjugation and the achieved TCO loading was studied within the range of 10–1000 eq. TCO (Figure 2C). A maximum of 2.6 TCOs/NB was achieved with TCO-PEG_4_-NHS using 100 eq., whereas a maximum of 1.5 TCOs/NB was reached with TCO-NHS ester using 250 eq. Higher TCO equivalents resulted in lower TCOs per NB ratios (Figure 2C, left). A possible explanation for this observation could be that high conjugation levels of the relatively lipophilic TCOs result in lower NB solubility and protein aggregation. These NBs would be difficult to titrate and result in an underestimation of TCOs per NB. We base this assumption on the fact that at higher TCO equiv. additions, the reaction solution shifted from a clear solution to a white suspension, especially when we applied 1000 eq.

In the following step, we estimated the TCO-loading per NB needed to label the NB at microdose levels. Our calculations showed that a TCO/NB load of approximately 0.2 was required to be able to label our NBs at microdose levels (see Supplementary Materials). These calculations are heavily dependent on the molar activity (A_m_). Since A_m_ can vary significantly between batches of ^18^F-labeled tetrazine and between production sites, we decided to use a ratio of approximately 1 TCO/NB to include a 5-fold safety margin while still keeping the level of protein modifications low. To minimize possible alterations in the physicochemical properties of our NB (W25), we selected the smallest linker length between our TCO and the NB. Consequently, only TCO-NBs conjugated *via* TCO-NHS ester were used for further experiments. The batch used for the following experiments contained 0.8 TCO/NB.

In contrast to W25, W25Fc is a larger protein (approx. 80 kDa compared to the 16 kDa NB). Therefore, we aimed to conjugate a higher amount of TCO to this NB construct. We considered the linker length between the TCO and the NB construct less relevant and therefore used the TCO-PEG_4_-NHS ester for conjugation. Additionally, a longer linker results in higher reactivity of conjugation points when large proteins such as W25Fc are labeled – without significantly altering the protein properties (44). TCO-W25Fc was tagged with approximately 9 TCO/Ab and used for further evaluation.

### In vitro binding to spike protein

**Figure 3:**
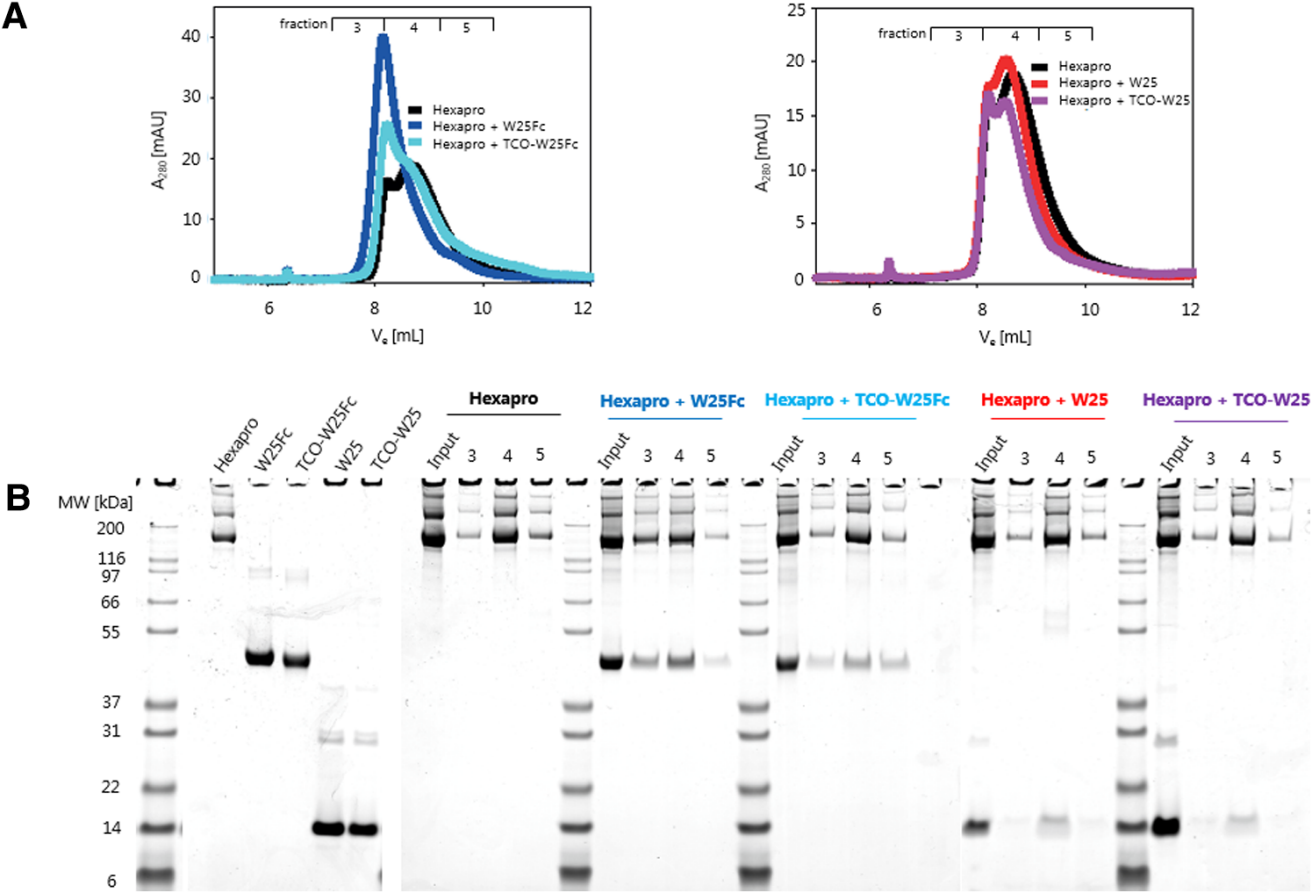
(**A**) FPLC analytical GF traces of hexapro (spike protein) and hexapro incubated with TCO-W25 and TCO-W25Fc. Samples: ∼10 μM Hexapro + ∼30 μM W25 of ∼10 μM W25Fc, in a volume of 100 μl incubation on ice. SEC: Superdex 200 increase 10/300 GL. Buffer: 10 mM Tris-HCl pH 7.8, 150 mM NaCl, 0.5 ml/min, 1 ml fractions (**B**) SDS-PAGE results of TCO-W25 and TCO-W25Fc incubated with Hexapro. In vivo PET in rats.

TCO-W25 and TCO-W25Fc were incubated with SARS-CoV2 Spike protein (Hexapro variant (55)) to evaluate their binding potential to Spike using analytical gel filtration (GF). Unmodified W25 and W25Fc were used as controls. All samples were loaded on an analytical GF column (Superdex 200 Increase 10/300 GL, Cytiva), and 1 ml fractions were collected. The GF fractions were evaluated by SDS-PAGE, and the binding of both TCO-W25 and TCO-W25Fc to the Spike protein was confirmed by coelution of the proteins, concomitant with a shift of the GF elution peak to higher molecular weight (Figure 3).

### Radiolabeling of TCO-W25 and TCO-W25Fc

Tz**1** was chosen for radiolabeling of the modified proteins due to its relatively high stability and the possibility to produce this Tz at large scale with high A_m_. ^18^F-labeling of Tz**1** and Tz**2** was performed as previously described (SI) (49). [^18^F]Tz**1** was incubated with an excess of TCO-W25 (2 equiv.) to provide [^18^F]**1**W25 with a radiochemical purity (RCP) >95% without requiring a purification step after the click reaction. [^18^F]Tz**2** was incubated with a lower excess of TCO-W25 (1.4 equiv.), which resulted in a radiochemical conversion (RCC) of 78%. Therefore, a final purification step with a PD10 column was required to obtain [^18^F]**2**W25 with a RCP >96%. Radiolabeling of [^18^F]**1**W25Fc could be achieved without purification, using 3 eq. of TCO-W25Fc (RCP > 95%). All tracers were formulated in PBS with an activity concentration between 15 and 25 MBq/ml and used for PET studies in rats. The feasibility of microdosing was tested for [^18^F]**1**W25. Radiolabeling of [^18^F]**1**W25 resulted in a specific activity of 2.5 MBq/μg of protein, indicating that for a human dose of 300 MBq, a total of approximately 122 μg [^18^F]**1**W25 is needed. This is higher than the microdosing requirements of <100 μg NB per patient, but it is on the same order of magnitude. Lowering the injected dose per patient to 200 MBq would be considered microdosing since only 81.6 μg of protein would be required and injecting 250 MBq would require approximately 100 μg of protein.

**Figure 4:**
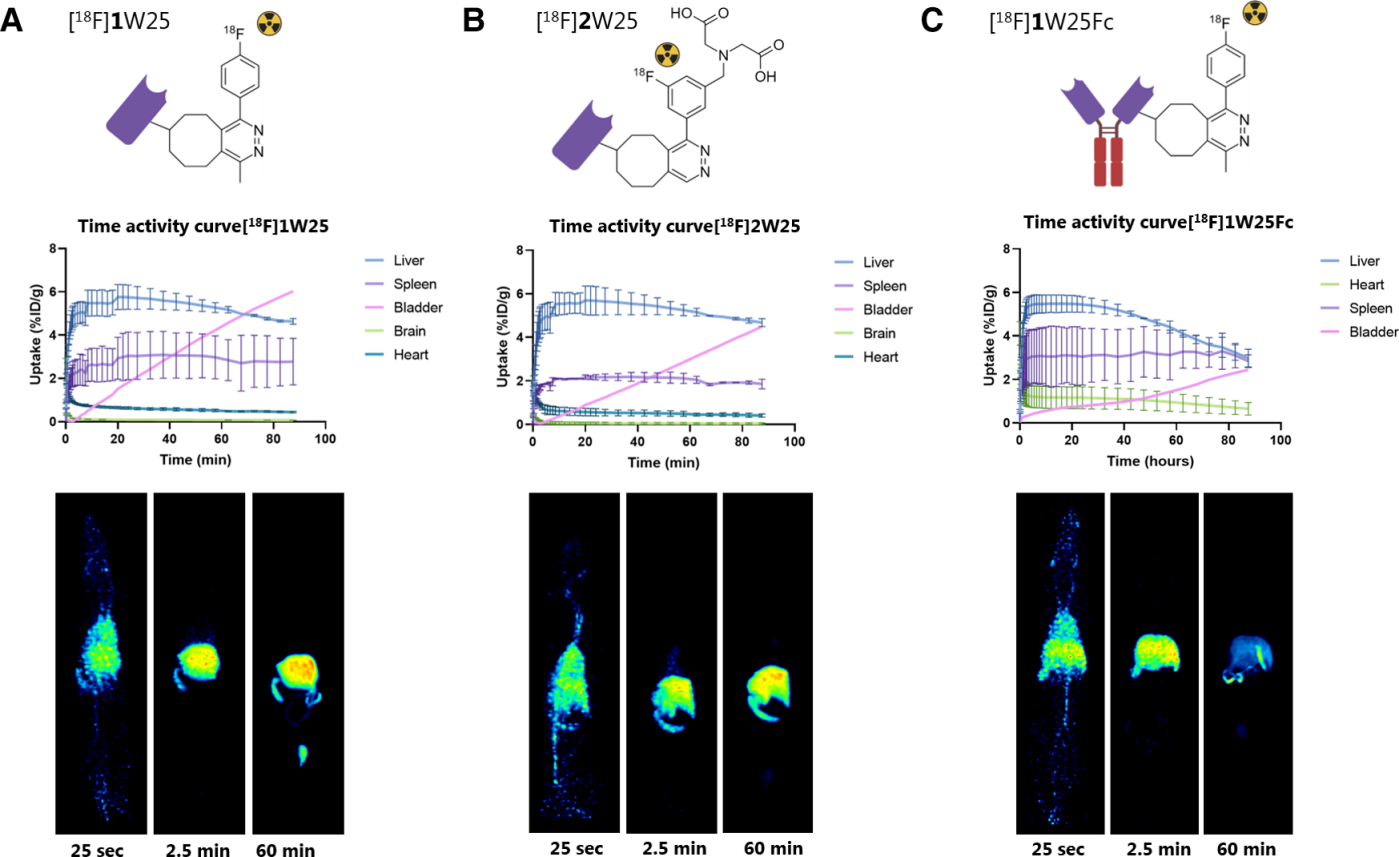
Time activity curves (TACs) and examples of PET images of 90 min PET scans in rats with (**A**) [^18^F]**1**W25, (**B**) [^18^F]**2**W25 and (**C**) [^18^F]**1**W25Fc. Excretion through the liver was observed for all three compounds.

The ^18^F-labeled proteins were injected intravenously in Long Evans rats (10–20 MBq per animal) and scanned for 90 min in a PET scanner to evaluate the biodistribution of the compounds. Activity uptake in key excretion organs was quantified. Additionally, brain uptake was quantified for W25 NB, since – even though not common - some NBs are known to cross the blood-brain barrier (BBB) (56). However, the PET data revealed no significant brain uptake of our NB. PET scanning with [^18^F]**1**W25 showed rapid excretion through the liver, whereas NBs, in most cases, are excreted through the kidneys (Figure 4A) (57). We hypothesized that this could be due to the lipophilicity of the labeled NBs resulting from adding the TCO-Tz complex. To test this, we labeled TCO-W25 with a more polar Tz. [^18^F]**2**W25 was also evaluated in a 90 min PET scan (Figure 4B). Similar biodistribution profiles were observed, implying that the polarity, charge and size of the Tz does not significantly impact the biodistribution. Even though excretion through the kidneys is more common for NBs, liver excretion was not considered a major concern since this does not overlap with the region of interest. Since we could not find an explanation for this unexpected liver excretion, we decided to radiolabel and evaluate an Fc-fused NB (W25Fc) targeting Spike protein. This 80 kDa protein is significantly larger than the NB (approx. 16 kDa). Therefore, we expect the TCO-modifications and click reaction to interfere less with the protein's properties and *in vivo* behavior. As expected for larger proteins, [^18^F]**1**W25Fc also showed excretion mainly through the liver (Figure 4C). None of our regions of interest - the lungs and airways - showed uptake of the radioligands, as expected for WT animals and thus implying that we could see a difference in imaging contrast in SARS-CoV-2 animal models (57).

## Conclusion

In this work, we describe the development of SARS-CoV-2 Spike targeted nanobody-based PET tracers. For this, we used NB W25 and W25Fc. The tetrazine ligation was successfully used to ^18^F-label both NBs with an RCP >95%. Both proteins were able to bind to the Spike protein *in vitro*. Dynamic PET scans over 90 min showed that W25 and W25Fc are mainly excreted in the liver of rats, and no uptake was observed in the lungs and airways. Microdosing can be achieved for human doses of 250 MBq per patient. However, a dose of 300 MBq per patient would be preferred, which might be possible with further optimization of the tetrazine ligation reaction. We plan to carry out future studies showing that the developed NBs can bind to the Spike protein *in vivo*. Since the start of the pandemic in 2019, several COVID-19 animal models have been developed, which could be helpful for our studies (58, 59). In vivo Spike binding can therefore be conducted in mice or WT rats injected with Spike protein. We aim to use one of these models to evaluate the binding of our radioligands to Spike *in vivo*.

## Data Availability

The original contributions presented in the study are included in the article/**Supplementary Material**, further inquiries can be directed to the corresponding author/s.
