# Supplementary material for: Development and evaluation of an 18F-labeled nanobody to target SARS-CoV-2's spike protein
Source: Front Nucl Med. 2022 Nov 23;2:1033697. doi: 10.3389/fnume.2022.1033697 (PMC11440877; doi:10.3389/fnume.2022.1033697)
Supplement: Supplementary file 1 [file Datasheet1.docx]

Supporting Information

Table of Contents

[Section S1: Cell assay 2](#_Toc112856344)

[Cell Culture 2](#_Toc112856345)

[High content microscopy 2](#_Toc112856346)

[Section S2: TCO-modifications 2](#_Toc112856347)

[TCO-modifications W25 with TCO-PEG_4_-NHS ester 2](#_Toc112856348)

[TCO-modifications W25 with TCO-NHS ester 2](#_Toc112856349)

[Scale-up of TCO-modifications W25 with TCO-NHS ester 2](#_Toc112856350)

[TCO-modifications W25Fc with TCO-PEG_4_-NHS 3](#_Toc112856351)

[Section S3: TCO titration by SDS-PAGE with ^111^In-tetrazine 3](#_Toc112856352)

[Section S4: Binding Assay 3](#_Toc112856353)

[Section S5: Radiochemistry 3](#_Toc112856354)

[Materials 3](#_Toc112856355)

[General information 3](#_Toc112856356)

[Radiolabeling of Tz [^18^F]**1** 4](#_Toc112856357)

[Radiolabeling of Tz [^18^F]**2** 4](#_Toc112856358)

[Radiolabeling TCO-W25 with Tz [^18^F]**1** 5](#_Toc112856359)

[Radiolabeling TCO-W25 with Tz [^18^F]**2** 5](#_Toc112856360)

[Radiolabeling TCO-W25Fc with Tz [^18^F]**1** 6](#_Toc112856361)

[Preparation of of Tz [^111^In]**3** 6](#_Toc112856362)

[Section S6: In vivo PET 7](#_Toc112856363)

[Animals 7](#_Toc112856364)

[PET procedure 7](#_Toc112856365)

[Reconstruction and quantification of PET data. 7](#_Toc112856366)

[Section S7: Calculation of the minimum required TCO 7](#_Toc112856367)

[References 8](#_Toc112856368)

# Section S1: Cell assay

## Cell Culture

H1299 cells were maintained at 37°C in RPMI supplemented with 10% FCS and 100 units/mL of penicillin and streptomycin. The vector GFP-tagged full length Spike of SARS-CoV-2 (Wuhan) was transfected in 10 cm plates using 10 µg of DNA, 24h after transfection cells were split into 96 well plate ~10000 cells per well. Transfection was performed using Lipofectamine 2000 (Invitrogen) according to the manufacturer’s instructions and media were supplemented with Normocin during transfection (Invivogen).

## High content microscopy

Spike-GFP transfected H1299 cells were grown on a 96-well optical plate (Themofisher). The H1299 cells were either observed directly under the microscope, Celldiscoverer 7 (Carl Zeiss GmbH, Jena, Germany) or incubated for 1h with Myc tagged W25 (1µg/mL) in the culture media and washed with PBS (3x) and fixed with 4% paraformaldehyde at room temperature for 30 min. After fixation, cells were washed with PBS and permeabilized in PBS 0.2% TritonX100. After washing the cells in PBS (3x), cells were incubated during 45 minutes at 37°C with a mouse anti-myc antibody (Cell Signaling) at a ratio 1:3000. Directly afterward, the cells were washed in PBS (3x) and incubated with an anti-mouse Alexa 647 Ab during 35 minutes at 37°C. For nuclei staining, cells were washed with PBS and incubated for 10 min at room temperature with DAPI (0.1 mg/mL). After the final wash, cells were maintained in the 96-well optical plates in PBS (100 µL). Images of fixed cells were acquired with a high content automatic microscope, Celldiscoverer 7 (Carl Zeiss GmbH, Jena, Germany).

# Section S2: TCO-modifications

## TCO-modifications W25 with TCO-PEG_4_-NHS ester

100 μL of W25 (0.15 mg/mL, 31.6 μM) in PBS (pH 7.4) was aliquoted. For the different aliquots, we added varying amounts, 10-1000 equiv., of TCO-PEG_4_-NHS ester (Broadpharm, BP-22418) and sodium carbonate buffer (1 M, 3.2 μL, pH 8.0) to reach a final concentration of 30 mM. The mixture was incubated at 600 rpm for 2 hours at room temperature in the dark. Unreacted TCO-PEG_4_-NHS ester was removed by purification with Zeba spin desalting columns (7K MWCO, 0.5 mL, 89882, Thermofisher). The final protein concentration was measured with a Nanodrop (NanoDrop 2000, ThermoScientific).

## TCO-modifications W25 with TCO-NHS ester

100 μL of W25 (0.15 mg/mL, 31.6 μM) in PBS (pH 7.4) was aliquoted in two vials. For the different aliquots, we added varying amounts, 10-1000 equiv., of TCO-NHS ester (Broadpharm, BP-22417) and sodium carbonate buffer (1 M, pH 8.0) was added to reach a final concentration of 30 mM. The mixture was incubated at 600 rpm for 2 hours at room temperature in the dark. Unreacted TCO-PEG_4_-NHS ester was removed by purification with Zeba spin desalting columns (7K MWCO, 0.5 mL, 89882, Thermofisher) and eluted in PBS (pH 7.4). The final protein concentration was measured with Nanodrop (NanoDrop 2000, ThermoScientific).

## Scale-up of TCO-modifications W25 with TCO-NHS ester

50 μL aliquots containing different W25 concentrations, ranging from 0.15-1.0 mg/mL, were prepared. For each aliquot, we added a 80 equiv. of TCO-NHS ester (Broadpharm, BP-22417) and sodium carbonate buffer (1 M, pH 8.0) to reach a final concentration of 30 mM. The mixture was shaken at 600 rpm for 2 hours at room temperature in the dark. Unreacted TCO-NHS ester was removed by purification with Zeba spin desalting columns (7K MWCO, 0.5 mL, 89882, Thermofisher) and eluted in PBS (pH 7.4). The final protein concentration was measured with a Nanodrop (NanoDrop 2000, ThermoScientific).

## TCO-modifications W25Fc with TCO-PEG_4_-NHS

100 μL of W25Fc (2.0 mg/mL) in PBS (pH 7.4) was aliquoted in two vials. For each aliquot, we added a 400 equiv. of TCO-PEG_4_-NHS (Broadpharm, BP-22418) and sodium carbonate buffer (1 M, 5.35 μL, pH 8.0). The mixture was shaken at 600 rpm for 2 hours at room temperature in the dark. Unreacted TCO-PEG_4_-NHS was removed by purification with Zeba spin desalting columns (7K MWCO, 0.5 mL, 89882, Thermofisher) and eluted in PBS (pH 7.4), resulting in a >95% of protein recovery. The final protein concentration was 2.5 mg/mL, measured with a NanoDrop (NanoDrop 2000, ThermoScientific). Quantification by radioactive SDS-PAGE (SI, Section S2) revealed the presence of approximately 11 reactive TCO/protein.

# Section S3: TCO titration by SDS-PAGE with ^111^In-tetrazine

Titration experiments were conducted to quantify the amount of reactive TCOs per protein-conjugate using a previously reported procedure.(1) We mixed aliquots of TCO-mAb in PBS (5 µL of 0.1-1.0 mg/mL solutions) with aliquots of ^111^In-labeled Tz ([^111^In]**1)** stock (5 µL, SI, Section S4) containing 2-3 eq. of Tz per expected amount of TCOs on the TCO-mAbs, and the mixed samples were incubated at 600 rpm for 1 hour at 37 °C. 3 μL of NuPAGE™ LDS Sample Buffer (NP0007, Invitrogen) was added and the mixture and shaken for 10 minutes at 70 °C. Samples were applied to NuPAGE™ 4 to 12%, Bis-Tris, 1.0 mm, Mini Protein Gel, 12-well (NP0322BOX , Invitrogen) SDS-PAGE gels. SDS-PAGE gels were exposed to phosphor storage screens and read by a Cyclone Storage Phosphor System (PerkinElmer Inc.). Quantification of plate readings was done with Optiquant software (version 5.00, PerkinElmer Inc.) based on a previously reported procedure.(1)

# Section S4: Binding Assay

10 µM of SARS-CoV2 Spike protein (Hexapro variant) was incubated with 30 µM W25/TCO-W25 or 10 µM W25Fc/TCO-W25Fc in 100 µL of buffer containing 10 mM Tris-HCl (pH 7.8) and 150 mM NaCl for 12 h on ice. Samples were loaded on an analytical gel filtration column (Superdex 200 Increase 10/300 GL, Cytiva), equilibrated in 10 mM Tris-HCl pH 7.8, 150 mM NaCl, and eluted at a flow rate of 0.5 mL/min, using an Äkta pure FPLC (Cytiva). 1 mL fractions were collected and analyzed by SDS-PAGE.

# Section S5: Radiochemistry

## Materials

Unless otherwise stated, all reagents and solvents were purchased from commercial suppliers and used without further purification. All the water used was ultrapure (> 18.2 MΩ cm-1). Other solvents were analytical or HPLC grade, and were used as received.

## General information

Radiochemistry was performed at the Department of Clinical Physiology, Nuclear Medicine and PET, Rigshospitalet, Denmark. [^18^F]Fluoride was produced by the (p,n) reaction in a cyclotron (60 mikroA CTI Siemens or 40 mikroA Scanditronix) by irradiating [^18^O]H_2_O with a proton beam of 11 MeV (CTI siemens) or 16 MeV (Scanditronix ). [^111^In]InCl_3_ was purchased from Curium. Automated syntheses were performed in a Scansys Laboratorieteknik synthesis module housed in a hot cell. The analytical-HPLC system consists of a 170U UVD detector, a Scansys radiodetector and a Dionex system connected to a P680A pump. The system was run by Chromeleon software. The used HPLC columns, solvent system and methods for radio-HPLC analysis are stated below for each compound.

The radiochemical conversion (RCC) of the radiolabeled compounds was determined by analyzing an aliquot of the crude reaction mixture by radio-HPLC analysis integrating the radioactive peaks of the chromatogram.(2) Radiolabeled products were characterized by associating the UV-HPLC traces of the authentic cold compounds with the radio-HPLC chromatogram of the reaction mixtures. The radiochemical yield (RCY) was determined using the initial activity at the beginning of synthesis and that of the formulated product at the end of synthesis (E.O.S.), corrected for decomposition and corrected for decay (d.c.). The molar activity (Am) was determined by integrating the area of the HPLC-UV absorbance peak of the radiolabeled product in the HPLC chromatogram. Radiochemical purity (RCP), radiochemical yield (RCY) and molar activity (Am) values are given as mean values. This applies to all radiolabeled compounds described below.

Radiochemical conversion (RCC) of all radiolabeled compounds was determined by analyzing a labeled aliquot of the reaction mixture by radio-HPLC and analyzed by integrating the radioactive peaks from the reaction solution. The products were characterized by comparing the radio-HPLC trace of the reaction mixtures with the HPLC UV traces of the authentic ^19^F-reference samples, respectively. The radiochemical yield (RCY) was determined using the [^18^F]fluoride activity received from the cyclotron at the beginning of the synthesis and that of the formulated product at the end of the synthesis, the decomposition was corrected and have been decay corrected (d.c.). The molar activity (Am) was determined by integrating the area of the UV absorbance peak corresponding to the radiolabeled product on the HPLC chromatogram. The area of the UV absorbance peak corresponding to the radiolabeled product was measured (integrated) on the HPLC chromatogram. This value was then converted into a molar concentration by comparison with an average integrated area of a known standard of the reference compound (triplicate)

## Radiolabeling of Tz [^18^F]**1**

Reference compound **1** was prepared as described previously.(3) [^18^F]**1** was radiolabeled from the precursor 3-(4-trimethyltin)-6-methyl-1,2,4,5-tetrazine (**1b**) as previously described by García-Vázquez et al. (Scheme S1).(3) Tz [^18^F]**2** was afforded in a RCY (d.c.) of 25±7% , a RCP ≥ 98% and A_m_ of 190 ± 10 GBq/μmol (d.c) (n=6). A typical activity yield was 2.5–3 GBq starting from ~12 GBq fluoride-18, and the total duration of the synthesis was 1 hour.

*Scheme S1:* *Schematic overview to synthesize Tz precursors (1b), Tz reference compounds (1) and radiolabeled ^18^F-Tz ([^18^F]1). i) MeCN, Zn(OTf)2, NH2NH2 . H2O, EtOH, 60 ºC, 24 h; ii) NaNO2, AcOH, 0 ºC, 20 min, 39% (1), 20% (4), 30% (6); iii) (Me3Sn)2, Pd(OAc)2, meCgPPh, THF, 70 ºC, MW, 30 min, 76%; iv) B2pin2, Pd2dba3, XPhos, KOAc, 1,4-Dioxane, 24 h, 110ºC, 63%; v) Cu(OTf)2, pyridine, [^18^F]KF, DMA, 5 min, 100 °C.*

## Radiolabeling of Tz [^18^F]**2**

Reference compound **2** was prepared according as previously described.(3) [^18^F]**2** was radiolabeled from the precursor Di-tert-butyl 2,2'-((3-(1,2,4,5-tetrazin-3-yl)-5 (trimethylstannyl)benzyl)azanediyl) diacetate (**2d**) as previously described by Garcia-Vazquez et al (Scheme S2).(3) Tz [^18^F]**2** was afforded in a RCY (d.c.) of 11±5%, a RCP ≥ 98% and A_m_ of 138 ± 13 GBq/μmol (d.c) (n=6). A typical activity yield was 600–700 MBq starting from ~12 GBq fluoride-18, and the total synthesis duration was 90 minutes.

Scheme S2: Synthesis of Tzs [^18^F]**2** (a) and **2** (b). **(a)** i) NBS, AIBN, CHCl_3_, 65 ºC, 24 h; 49%; ii) K_2_CO_3_, MeCN, 25 ºC, 24 h, 99 %; iii) DCM, S_8_, NH_2_NH_2_ ^.^ H_2_O, EtOH, 50 ºC, 24 h, 15–24%; iv) (Me_3_Sn)_2_, Pd(PPh_3_)_4_, THF, 65 ºC, MW, 3 h, 62%; v) Cu(OTf)2, pyridine, [^18^F]KF, DMA, 5 min, 100 °C ; vi) TFA, MeCN, 100 ºC, 15 min ; **(b)** i) NBS, AIBN, CHCl_3_, 65 ºC, 12 h, 52%; ii) K_2_CO_3_, MeCN, r.t., 24 h, 89%; iii) DCM, S_8_, NH_2_NH_2_ ^.^ H_2_O, EtOH, 50 ºC, 24 h, 17%; iv) TFA, DCM, 25 ºC, 2 h, 51%.

## Radiolabeling TCO-W25 with Tz [^18^F]**1**

TCO-W25 (425 μL, 10.2 nmol protein, 8.17 nmol TCO) and Tz [^18^F]**1** (RCP = 98%, 400 MBq, 500 μL, 4.09 nmol, 0.5 TCO/Tz equiv.) were added to a 5 mL Eppendorf tube. The mixture was shaken at 600 rpm for 30 min at 37 °C to give [^18^F]**1** W25 with a RCP of > 95% as confirmed by radio-HPLC (Aeris™ 3.6 µm Widepore C4 200 Å, LC Column 150 x 4.6 mm, Solvent A = 0.1% TFA in water, solvent B = 0.1% TFA in acetonitrile. HPLC elution method: 0-4.5 min – 25% B, 4.5-8 min - gradient from 25% B to 90% B, 8-10.5 min – 90% B, 10-12.5 min - back to 25% B, 12.5-15 min – 25% B; flow rate 1.5 mL/min). The radiotracer was formulated in PBS to obtain a final activity concentration of 24.5 MBq/mL (n.d.c) and was used for animal experiments.


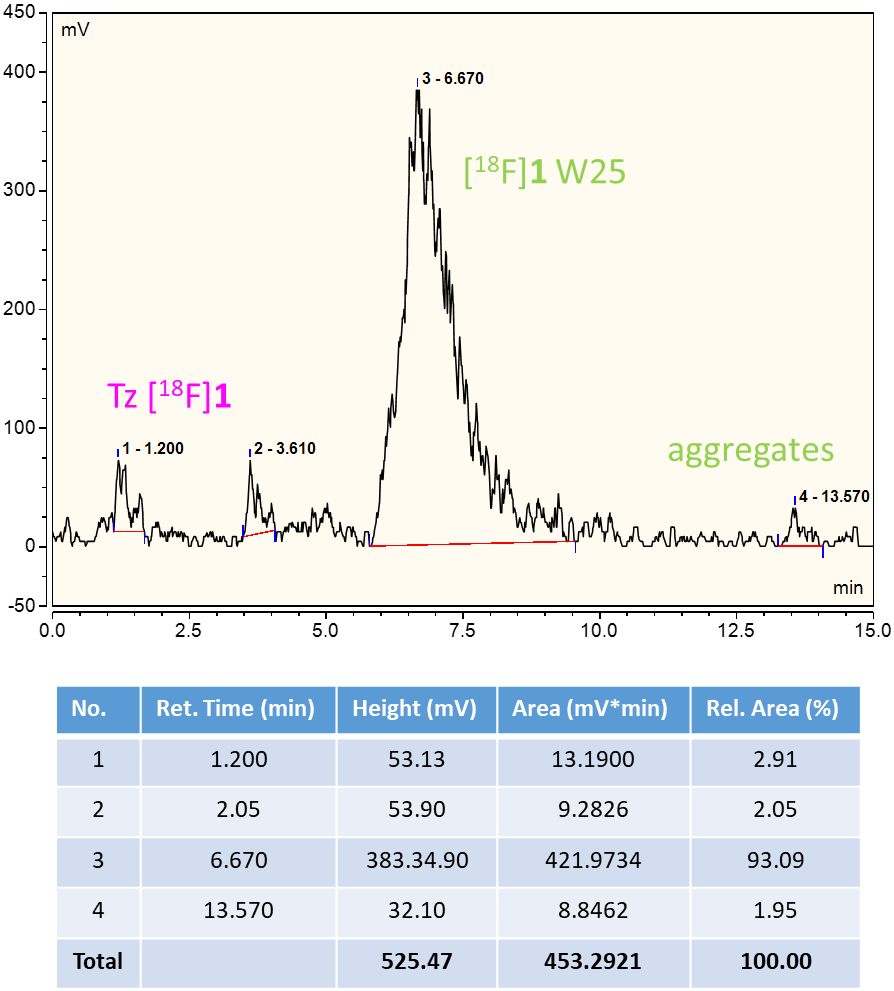


Figure 1: RadioHPLC chromatogram of [^18^F]**1** W25. Aeris™ 3.6 µm Widepore C4 200 Å, LC Column 150 x 4.6 mm, Solvent A = 0.1% TFA in water, solvent B = 0.1% TFA in acetonitrile. HPLC elution method: 0-4.5 min – 25% B, 4.5-8 min - gradient from 25% B to 90% B, 8-10.5 min – 90% B, 10-12.5 min - back to 25% B, 12.5-15 min – 25% B; flow rate 1.5 mL/min

## Radiolabeling TCO-W25 with Tz [^18^F]**2**

TCO-W25 (250 μL, 6.0 nmol protein, 4.81 nmol TCO) and Tz [^18^F] **2** (RCP =91%, 90 MBq, 1000 μL, 4.09 nmol, 0.5 TCO/Tz eq.) were added to a 5 mL Eppendorf tube. The mixture was shaken at 600 rpm for 30 min at 37 °C. Purification was carried out using a PD-10 desalting column (Cytiva) to give [^18^F]**2** W25 with a RCP of 96% as confirmed by radio-HPLC (Aeris™ 3.6 µm Widepore C4 200 Å, LC Column 150 x 4.6 mm, Solvent A = 0.1% TFA in water, solvent B = 0.1% TFA in acetonitrile). HPLC elution method: 0-4.5 min – 25% B, 4.5-8 min - gradient from 25% B to 90% B, 8-10.5 min – 90% B, 10-12.5 min - back to 25% B, 12.5-15 min – 25% B; flow rate 1.5 mL/min). A total amount of 56 MBq [^18^F]**2** W25 was recovered (12.7 MBq/mL PBS (n.d.c)) and used for the animal experiments.


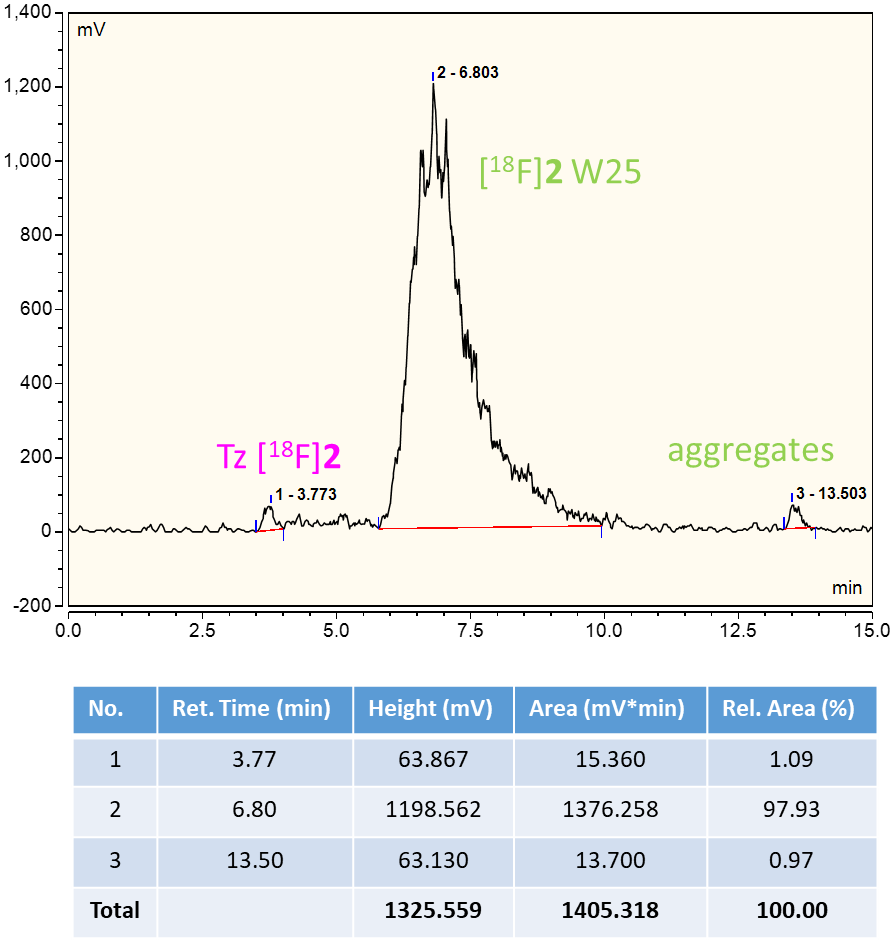


Figure 2: RadioHPLC chromatogram of [^18^F]**2** W25. Aeris™ 3.6 µm Widepore C4 200 Å, LC Column 150 x 4.6 mm, Solvent A = 0.1% TFA in water, solvent B = 0.1% TFA in acetonitrile). HPLC elution method: 0-4.5 min – 25% B, 4.5-8 min - gradient from 25% B to 90% B, 8-10.5 min – 90% B, 10-12.5 min - back to 25% B, 12.5-15 min – 25% B; flow rate 1.5 mL/min

## Radiolabeling TCO-W25Fc with Tz [^18^F]**1**

TCO-W25Fc (30 μL, 101.86 nmol protein, 9.26 nmol TCO) and Tz [^18^F] [^18^F]**1** (RCP = 98%, 100 MBq, 887 μL, 2.78 nmol, 0.3 TCO/Tz eq.) were added to a 5 mL Eppendorf tube. The mixture was shaken at 600 rpm for 30 min at 37 °C, yielding [^18^F]W25Fc in a RCP of > 95% as confirmed by radio-HPLC (Aeris™ 3.6 µm Widepore C4 200 Å, LC Column 150 x 4.6 mm, Solvent A = 0.1% TFA in water, solvent B = 0.1% TFA in acetonitrile. HPLC elution method: 0-4.5 min – 25% B, 4.5-8 min - gradient from 25% B to 90% B, 8-10.5 min – 90% B, 10-12.5 min - back to 25% B, 12.5-15 min – 25% B; flow rate 1.5 mL/min). The radiotracer was formulated in PBS to a final activity concentration of 18.7 MBq/mL (n.d.c) and used for animal experiments.


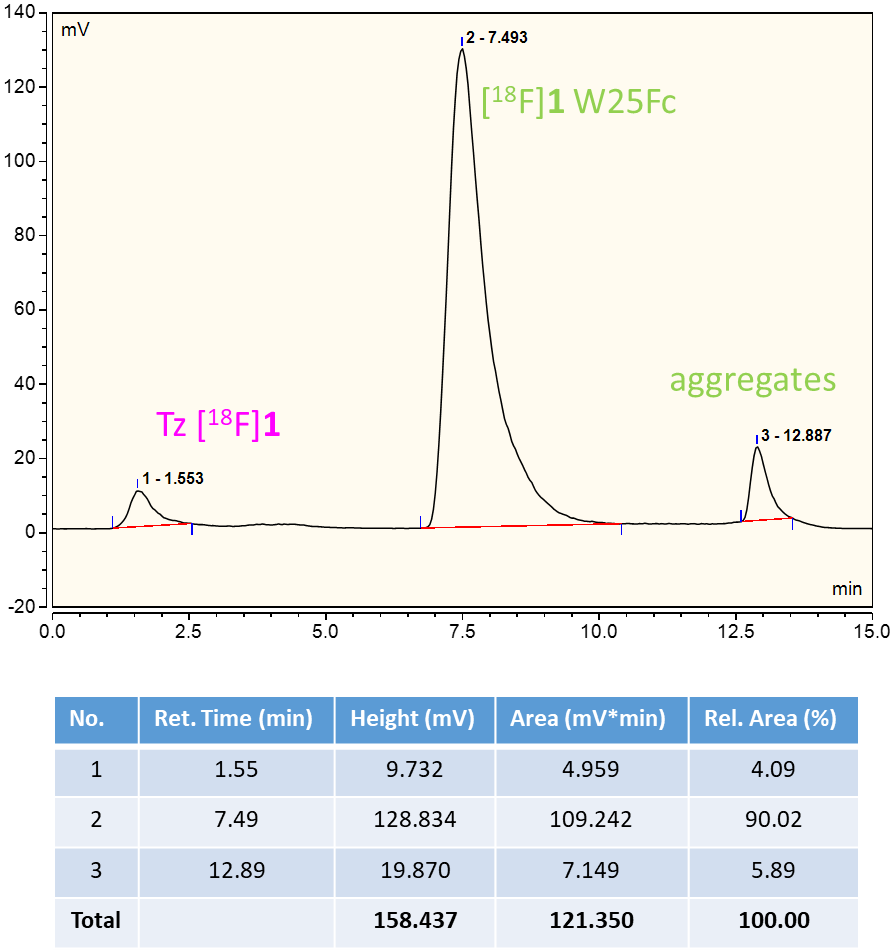


Figure 3: RadioHPLC chromatogram of [^18^F]**1** W25Fc. Aeris™ 3.6 µm Widepore C4 200 Å, LC Column 150 x 4.6 mm, Solvent A = 0.1% TFA in water, solvent B = 0.1% TFA in acetonitrile). HPLC elution method: 0-4.5 min – 25% B, 4.5-8 min - gradient from 25% B to 90% B, 8-10.5 min – 90% B, 10-12.5 min - back to 25% B, 12.5-15 min – 25% B; flow rate 1.5 mL/min

## Preparation of of Tz [^111^In]**3**

^111^In-labeled Tz was used for the titration of TCO-modified W25 and W25Fc (SI, Section S1)

The ^111^In-labeling was performed as previously described.(1) Briefly, **3** was dissolved (2 mg/mL) in metal-free water and stored at -80 °C before use. An aliquot of 50-100 μL (10-30 MBq) of [111In]indium chloride in 0.05 M HCl was combined with 2 μL DOTA-PEG_11_-tetrazine and 1 M NH_4_OAc buffer (pH 5.5) at a volume ratio of 1:10. The mixture was shaken at 600 rpm for 5 min at 60 °C in an Eppendorf ThermoMixer C. Then, 10 mM diethylenetriamine-pentaacetic acid DTPA (volume ratio 1:10) and 2 μL 10 mg/mL gentisic acid in saline was added, and the solution was shaken for an additional 5 min at 60 °C in an Eppendorf ThermoMixer C. Typically, a quantitative labeling yield and a radiochemical purity (RCP) > 95% were obtained with this method, as confirmed by radio-HPLC (Aeris Peptide C18-XB 3.6 µm 150x4.6 mm column. Solvent A = 0.1% TFA in water, solvent B = 0.1% TFA in acetonitrile. HPLC elution method: 0-1 min – 5% B, 1-8 min - gradient from 5% B to 75% B, 8-9 min – 75% B, 9-9.5 min - back to 5% B, 9.5-10 min – 5% B; flow rate 1.5 mL/min).

*Scheme S3. Preparation of [^111^In]3. i) ^111^InCl, 1M NH4OAc, 60 ºC, 5 min ii) 10 mM DTPA, gentisic acid, 60 ºC, 5 min*

# Section S6: In vivo PET

## Animals

Long-Evans WT female rats of 200–300 g (Charles River, Calco, Italy), were housed in groups of 2–3 rats per cage. The C57BL/6J female mice weighing 20-23 g (Janvier, France, La Rochelle) were housed in cages of 7-8 mice per cage. All rodents were kept in a climate-controlled facility with a 12-hr light/dark cycle. The cages contained a biting stick, fed with a commercial breeding diet *ad libitum* (1310 FORTI- Avlsfoder, Brogaarden, Altromin International) and had access to water. All procedures were conducted following the European Commission’s Directive 2010/63/EU, FELASA and ARRIVE guidelines for animal research and, with approval from The Danish Council for Animal Ethics (license numbers 2017-15-0201-01283) as well as the Department of Experimental Medicine, University of Copenhagen.

## PET procedure

PET scans were performed on a Siemens HRRT (High-Resolution Research Tomography). The quality control was executed with a rod containing germaninum-68 before scanning. The rats were transported to the scanner 2 hours before the scan, and anaesthesia was induced 1 hour before with 3% isoflurane in oxygen flow. After 10 min of induction, four rats were placed in 2 x 2 plastic cylinders connected to ~2% isoflurane in oxygen and suction. Each rat was provided with Eye-crème (Ophtha, øjensalve neutral). The reflexes were tested before a BD Neoflon 24G catheter was placed in the dorsal or lateral tail vein, stabilized with medical tape, and flushed with a solution of 4 units/mL Heparin in saline. 10-20 MBq of the radiotracer was administered through the catheter. In the scanner, the rats were kept warm with a heating lamp. The HRRT scanner contains a radioactive source (^137^Cs) used to perform the transmission scan. The 90 min emission scan was started at the time of radiotracer injection (8.0-12.7 MBq/rat). After the scan, the rats were placed in their cage and transported back to the facilities after waking up.

## Reconstruction and quantification of PET data.

The images were reconstructed with ordinary Poisson 3D ordered subset expectation maximization with point spread function, containing a point spread algorithm, resulting in 207 planes of 256 x 256 voxels (1.22 x 1.22 x 1.22 mm). The transmission attenuation map was reconstructed using the maximum posterior algorithm. The 90 min emission PET scan was transformed into 35 dynamic frames (6 x 10, 8 x 30, 5 x 60, and 16 x 300 sec) and filtered with 0 mm.(4) The time−activity curves were calculated for the following volumes of interest (VOIs): liver, bladder, heart and lungs (combined). The outcome measure in the time−activity curves was calculated as the radioactive concentration in the VOI (in kBq/mL) normalized to the injected dose corrected for the animal weight (in kBq/kg), yielding standardized uptake values (g/mL). Quantification of the binding was performed in PMOD (version 3.9)

# Section S7: Calculation of the minimum required TCO

The minimum required TCO/NB were calculated based on the following requirements

- - Dosing for humans: 300 MBq
  - According to microdosing concept(5,6): <100 μg NB
  - Average molar activity (A_m_) from our facilities: 250000 MBq/umol *

Minimum required TCOs was calculated as described below

- - $nmol TCO NB=\frac{Dose (\mu g)}{Am}=\frac{100 \mu g NB}{15800 \mu g/umol}=5.618 nmol$
  - $nmol TCO NB when 1 TCO/NB=\frac{Dose (MBq)}{Am}=\frac{300 MBq}{250000 MBq/umol}=1.2 nmol$
  - $min. number of TCOs needed =\frac{nmol TCO NB when 1 TCO/NB}{5.618nmol TCO NB nmol}\frac{1.2 nmol}{5.618 nmol}\approx0.2 TCO per NB$

* Based on average productions at Department of Clinical Physiology, Nuclear Medicine and PET, Rigshospitalet, Denmark.

# References

1. Shalgunov V, Lopes van den Broek S, Andersen IV, Garcia Vazquez R, Raval N, Palner M, et al. Pretargeted Imaging Beyond the Blood-Brain Barrier. ChemRxiv. 2022;Cambridge(This content is a preprint and has not been peer-reviewed.).

2. Herth M, Ametamey S, Antuganov D, Bauman A, Berndt M, Brooks A, et al. On the consensus nomenclature rules for radiopharmaceutical chemistry - Reconsideration of radiochemical conversion. Nucl Med Biol. 2020;

3. García-Vázquez R, Battisti UM, Jørgensen JT, Shalgunov V, Hvass L, Stares DL, et al. Direct Cu-mediated aromatic18F-labeling of highly reactive tetrazines for pretargeted bioorthogonal PET imaging. Chem Sci. 2021;12(35):11668–75.

4. Shalgunov V, Xiong M, L’Estrade ET, Raval NR, Andersen I v., Edgar FG, et al. Blocking of efflux transporters in rats improves translational validation of brain radioligands. EJNMMI Res [Internet]. 2020;10(1). Available from: https://doi.org/10.1186/s13550-020-00718-x

5. Burt T, John CS, Ruckle JL, Vuong LT. Phase-0/microdosing studies using PET, AMS, and LC-MS/MS: a range of study methodologies and conduct considerations. Accelerating development of novel pharmaceuticals through safe testing in humans–a practical guide. Expert Opin Drug Deliv [Internet]. 2017;14(5):657–72. Available from: http://dx.doi.org/10.1080/17425247.2016.1227786

6. U.S. Department of Health and Human Services- Food and Drug Administration, Center for Drug Evaluation and Research (CDER). Microdose Radiopharmaceutical Diagnostic Drugs : Nonclinical Study Recommendations Guidance for Industry. Guideline [Internet]. 2018;(August):4. Available from: https://www.fda.gov/Drugs/GuidanceComplianceRegulatoryInformation/Guidances/default.htm
